# Two contrasting papers stimulate a commentary on the origins of tumour immunology, current cancer immunotherapies, and the future potential for cancer immunotherapy

**DOI:** 10.1038/s41416-022-02075-z

**Published:** 2022-12-07

**Authors:** Walter Bodmer

**Affiliations:** Weatherall Institute of Molecular Medicine, Department of Oncology, University of Oxford, Oxford, UK

**Keywords:** Immunogenetics, Cancer therapy

## Abstract

Two early papers expressing conflicting views on the occurrence of effective immune attack against cancers stimulate an analysis of the gradual development of an understanding of tumour biology. This understanding has led to the development of the strikingly effective check point blocking and CART anti-cancer immunotherapies, and the promise of more widely applicable therapies based on T cell attracting genetically engineered monoclonal antibodies.

Iris Hamlin [1] did a careful analysis of lymphocyte infiltration in a series of breast cancers and presented data that showed the prognosis for survival increased with the measured amount of lymphocyte infiltration. On this basis, she argued that “that the body has a defence mechanism against tumour invasion and spread, and that this defence has failed in patients who die with widespread metastasis.” She was making the case for anti-cancer immune surveillance as first suggested by Ehrlich in 1909, but now based on the then only recent discovery by Gowans and others of the key role played by lymphocytes in the immune response. Hewitt et al. [2], on the other hand, in an extensive study of 27 different sorts of spontaneous mouse tumours in “isogenic” recipients, including attempts at direct or passive tumour immunisation, found no evidence for spontaneous immune attack against the transplanted tumours and so argued against the notion of cancer immunosurveillance, which might otherwise be the basis for the development of anti-cancer immunotherapies. He made the clearly valid point that most previous studies along these lines had used cancers induced by chemicals or viruses, which had given rise to changes in the tumours that could be recognised by the immune system, in contrast to the situation for spontaneously derived tumours.

A key feature of experiments by Hewett et al. was the use of isogenic strains of inbred mice from which the spontaneous tumours were derived. Many of the early extensive experiments on transplantation of tumours in mice were carried out at the Imperial Cancer Research Fund (ICRF) at the beginning of the 1900s by the first two directors of the ICRF, without any awareness of the genetic heterogeneity of the mouse strains and the importance of that, and that it was the genetic heterogeneity and not the cancerous state, which accounted for all the rejections of tumours that they observed [3]. Already in 1910 Peyton Rous (discoverer of the first cancer causing virus, the Rous Sarcoma virus) had shown that normal tissues transplanted between mice were rejected to the same extent as tumours [4]. Little CC realised the importance of the underlying genetic heterogeneity of the mouse strains being used at that time and so, by several generations of brother/sister mating, produced inbred, isogenic, mouse strains, some of which, including C57 Bl, are still widely used. With his colleague Tyzzer, he then showed that transplantation of tumours within inbred strains overcame the inconsistencies of the earlier experiments at the ICRF and elsewhere [5]. The next extremely important step was the discovery by Peter Gorer of the mouse H2 system as a genetically determined blood group and his demonstration that it was the differences between the H2 dominantly inherited genetic variants of different inbred mouse strains that controlled the tumour rejection [6]. It was the discovery of H2, the mouse major histocompatibility system, that foreshadowed the discovery of the HLA system, the human equivalent of H2, and the demonstration of the importance of these major histocompatibility systems in the overall control of the immune response.

As the situation with respect to the relationship between the immune system and cancers came to be somewhat clarified by the pioneering work on tumour immunology of George and Eva Klein, Lloyd Old and others, the idea of immunosurveillance as a mechanism for detecting and eliminating cancers because they developed immunologically recognisable differences from normal tissue was again promoted by well known immunologists, including McFarlane Burnet, see e.g., refs. [7] and, later, George Klein [8]. The notion, however, that immune surveillance evolved to protect against cancer is not supportable since cancer for most of the period of human evolution has not been a selective driving force as it occurs almost entirely after the end of the reproductive period. The same almost certainly applies to all mammals, since it is only because of the human achievement of countering most of the earlier causes of death that cancer has over at most the last 200 hundred years become an important human disease. The correlation in humans between cancer incidence and age can be simply explained by the fact that the factors selected for to avoid ageing before the end of the reproductive period also happen to influence cancer development. Cancer is a concomitant of senescence, and not a primary contributor to senescence. It is strange that George and Eva Klein accepted this argument in their earlier very incisive discussion of immune surveillance against virus induced tumours [9] while not doing so in the later paper.

The first clear cut evidence for immune attack against human cancers was the observation, using the then recently developed monoclonal antibody technology, of the lack of expression of HLA Class I proteins on the surface of certain cancer derived cell lines [10]. Subsequent studies showed that loss of expression of HLA Class I proteins due to complete loss of expression of β2 microglobulin was relatively common in colorectal cancers that were mismatch repair deficient [11]. This is as would be expected given the relatively high expected frequency of novel somatic mutations due to the mismatch repair defect resulting in novel targets for immune attack. Rather prophetically, as will be explained below, the paper ends by saying “The challenge is to design therapies aimed specifically at cells with a mutator phenotype that have lost HLA expression”.

One approach to enhance immunotherapy would be to increase the efficacy of the cancer patient’s own immune system and so improve the attack against novel tumour antigens. A dramatic step in this direction was achieved by treatment with antibodies that block the exhaustion of T cells directed against cells carrying a high novel antigenic load. This exhaustion is part of the normal mechanism for preventing over activity of the immune response. This blocking or “immune check point” therapy has worked remarkably well but only for those cancers that carry a high mutational burden, such as lung cancer due to the mutational effects of smoking, skin cancer due to UV induced mutations and mismatch repair defective cancers, just as was suggested might be needed given the commonly observed loss of HLA Class I expression, suggesting strong immune attack against such cancers [12].

Given the apparent limitations of enhancing the patient’s own adaptive immune system’s attack against their cancers, the next way forward is to design specific engineered immune attacks against novel or relatively overexpressed determinants in cancers, not only on the surface of the cancer cells but also internal to the cells. Obvious examples of such targets are tissue-specific proteins such as PSMA in prostate cancer, CD19 in B-cell derived lymphomas, or ectopic cancer expression such as placental alkaline phosphatase that is expressed in 10–15% of many carcinomas. Targeting can be achieved using engineered T cells directed by antibodies against the targets. CAR-T cell therapy, has proved very effective against haematological cancers but so far not against the much more common adenocarcinomas [13]. Another approach is to engineer monoclonal antibodies directed against a cancer target and also against T cells, by, for example, CD3. These antibodies can then bring T cells sufficiently close to the targeted tumour cells to kill them [14].

When the protein is only expressed inside the cell then we need a good so called Tcrm (T receptor mimic antibody), which mimics the T-cell receptor mechanism by recognising a peptide derived from the internal ectopically expressed protein when it is associated with an appropriate HLA type on the cell surface. This is not trivial to do but has been done.

Once you have good monoclonal antibodies for a cancer-specific appropriately expressed protein, they can easily be engineered into whatever immune killing system you choose. One key advantage of immune-based killing is that there is good evidence for bystander killing, namely killing by activated T cells of cancer cells in the vicinity of target carrying cancer cells, which do not express the target.

While there are still many hurdles in the way, monoclonal antibody mediated killing of cancer cells expressing ectopic proteins is, I believe, so far, the only sure way to get future cancer-specific-based treatments that can be coupled with check point blocking or other combinations. The key challenge is to find good new targets and, in order to avoid the development of resistance, not to attack only one target at a time.
